# Realization of the Kohn’s Theorem in Ge/Si Quantum Dots with Hole Gas: Theory and Experiment

**DOI:** 10.3390/nano9010056

**Published:** 2019-01-03

**Authors:** Hayk A. Sarkisyan, David B. Hayrapetyan, Lyudvig S. Petrosyan, Eduard M. Kazaryan, Anton N. Sofronov, Roman M. Balagula, Dmitry A. Firsov, Leonid E. Vorobjev, Alexander A. Tonkikh

**Affiliations:** 1 Institute of Engineering and Physics, Russian-Armenian University, Yerevan 0051, Armenia; dhayrap82@gmail.com (D.B.H.); edghaz@mail.ru (E.M.K.); 2 Institute of Physics, Nanotechnology and Telecommunications, Peter the Great St. Petersburg Polytechnic University, Saint Petersburg 195251, Russia; sofronov@rphf.spbstu.ru (A.N.S.); rmbal@spbstu.ru (R.M.B.); dmfir@rphf.spbstu.ru (D.A.F.); lvor@rphf.spbstu.ru (L.E.V.); 3 Department of Physics, Yerevan State University, Yerevan 0025, Armenia; 4 Department of Physics, Jackson State University, Jackson, MS 39217, USA; lyudvig1977@gmail.com; 5 OSRAM Opto Semiconductors, 93055 Regensburg, Germany; alexander.a.tonkikh@gmail.com

**Keywords:** quantum dots, Kohn theorem, adiabatic approximation, few-particle interaction, far-IR absorption, Ge/Si

## Abstract

This article discusses specific quantum transitions in a few-particle hole gas, localized in a strongly oblate lens-shaped quantum dot. Based on the adiabatic method, the possibility of realizing the generalized Kohn theorem in such a system is shown. The criteria for the implementation of this theorem in a lens-shaped quantum dot, fulfilled in the experiment, is presented. An analytical expression is obtained for the frequencies of resonant absorption of far-infrared radiation by a gas of heavy holes, which depends on the geometric parameters of the quantum dot. The results of experiments on far-infrared absorption in the arrays of p-doped Ge/Si quantum dots grown by molecular beam epitaxy (MBE) with gradually increasing average number of holes in dot are presented. Experimental results show that the Coulomb interaction between the holes does not affect the resonant frequency of the transitions. A good agreement between the theoretical and experimental results is shown.

## 1. Introduction

Quantum dots (QD) remain intensively studied objects of semiconductor nanostructure physics [1,2,3]. Since the spectrum of charge particles in QD can be flexibly controlled, the physical characteristics of such systems also become controllable. Significant role in the formation of the spectrum of charge carriers in QD is played by its geometry and sizes on the one hand, and the physicochemical characteristics of QD and the environment on the other [4]. Indeed, the energy spectrum of QD is determined by both the size and geometric form, and the component composition of the structure under study, as well as its environment. From the theoretical standpoint, the geometry of the QD identifies the symmetry of the Hamiltonian of the system, and the physicochemical characteristics of the structure under study form the profile of the confining potential of the QD. At the same time, the construction of a realistic Hamiltonian of the QD is extremely important, since this will provide the opportunity to bring the theory to the experiment.

Currently, QD of various geometric forms have been implemented: spherical [5,6], cylindrical [7,8], ellipsoidal [9,10], lens-shaped [11,12], ring-shaped [13,14], etc. Of these geometries, spherical and cylindrical QD have the simplest mathematical description, since in such cases the symmetry of the systems often allows the separation of variables in the corresponding Schrödinger equations [15,16,17]. However, for QD with a more complex geometry, even in the case of single-particle states, one has to use either approximate analytical or numerical methods [18,19,20].

At the first stage of the theoretical description of QD, it is necessary to determine the wavefunctions and the spectrum of charge carriers. Furthermore, based on the results obtained, one can proceed to the calculation of various physical parameters of the QD: absorption coefficient, relaxation time of particles, orbital current, etc. [21,22,23,24,25]. A particularly important role is played by the mathematical form of the confining potential of QD. When modeling the confining potential, it is necessary to take into account both the symmetry of the system under study and the mechanism of its growth. For example, if the growth method is such that it is necessary to take into account the effects of mechanical stress at the boundary of the QD—surrounding medium, then this circumstance should be considered when choosing the type of confining potential. This situation arises in the case of pyramidal QD [26,27]. On the other hand, if during the growth of QD, diffusion occurs between the components of the QD and the environment, then the profile of the confining potential is smoothed and it can be modeled by a parabolic potential or Wood-Saxon, Kratzer potential, etc. [28,29,30,31]. Special attention was paid to the parabolic confining potential. From a mathematical point of view, QD with a parabolic confining potential can be described analytically, including the case with the presence of external electric and magnetic fields [32,33,34,35]. At the same time, one of the most interesting and beautiful effects found in parabolic QD was the discovery of Kohn’s theorem in such systems [36,37,38,39,40]. Initially, this theorem was proved by the W. Kohn’s for the case of an electron gas in an external magnetic field [41]. A generalization to the case of a parabolic quantum well was given by the authors of reference [42]. Chakraborty and Maxim showed the implementation of this theorem in the 2D parabolic QD of circular section [36]. A similar problem for the case of two-dimensional asymmetric parabolic QD was considered by Peeters in reference [37].

The content of this theorem lies in the fact that the frequency of resonant absorption of longwave radiation from a pair-interacting electron gas localized in a parabolic QD doesn’t depend on the number of particles. In other words, single-particle transitions are realized in a multiparticle system. Mathematical proof is based on the possibility of representing the Hamiltonian of a pair-interacting gas as a sum of Hamiltonians, characterizing on one side the motion of the center of mass of the system, and on the other the relative motion of the gas under study. Since the incident perturbation depends only on the center of mass coordinates of the system, the resonant frequencies of the transitions don’t depend on the internal degrees of freedom, and therefore on the interparticle (in particular, Coulomb) interaction. Note that the parabolic profile of the confining potential is of fundamental importance, since in this case the variables of the center of mass and relative motion are separated.

As we mentioned above, the parabolic type of the confining potential can be formed due to the diffusion effect. However, a parabolic confinement may also arise due to the specific geometry of QD. In references [43,44,45], it was shown that for strongly oblate and prolated ellipsoidal QDs, the conditions for the realization of the generalized Kohn theorem can also be satisfied. At the same time, unlike other works, a confining potential doesn’t have a parabolic form initially: it is zero inside the QD and goes to infinity at the QD boundary. The specific form of the QD geometry allows the adiabatic method to be used to describe one-electron states in such systems [46].

As was shown in references [43,44], in the case of a strongly oblate ellipsoidal QD in the QD plane, the gas is localized in a two-dimensional parabolic quantum well. Given that size quantization in the *Z*-direction (the direction of compression of the ellipsoid) is much stronger than the Coulomb interaction between particles (electrons or holes), then this interaction can be considered effectively two-dimensional. Thus, the conditions for the generalized Kohn theorem can be fulfilled in such systems.

The possibility of controlling the parameters of QDs allows one to create nano- and optoelectronic devices with predetermined physical parameters. Among all possible material systems where the formation of quantum dots is possible, the MBE grown Ge dots in silicon matrix are of the great practical importance due to the compatibility with the well-developed silicon technology, in particular with the standard CMOS process [47]. Currently, most studies of structures with self-assembled MBE Ge/Si quantum dots in the silicon matrix are motivated by their wide potential applications in optoelectronics and are now divided into two large trends related to optical and photoelectric phenomena in the near infrared (IR) and mid infrared spectral regions. The electroluminescence and photoconductivity associated with interband transitions of charge carriers in quantum dots were observed in overlapping near-IR spectral regions below the silicon bandgap in silicon p-i-n structures with embedded Ge/Si QD layers in Refs [48,49].

Recent research [50,51,52,53,54] describes new experimental results on the mid-IR photoresponse of Ge/Si QDs that are important for instrumental applications. In particular, photoconductivity was observed in reference [50] under the conditions of electron delocalization, in reference [51] the phonon bottle-neck effect was discussed, in reference [52] the influence of structure doping on the characteristics of photodetectors was studied, and in references [53,54] the multifold enhancement of the intraband photocurrent of holes due to interaction with surface plasmons in a structure coated with a gold film with ordered nanoholes was experimentally revealed.

Recent advances in MBE growth technology demonstrate a significant increase in the surface density of the Ge/Si quantum dots in self-assembled arrays with help of surfactant-mediated epitaxy [55,56]. In this case, a thin layer of Sb remains on the growing surface during the entire growth process, and the size and shape of quantum dots can be controlled with the growth temperature and deposition rate. This method allows one to obtain the quantum dots in the form of Ge (or GeSi solid solution) islands in the silicon matrix with the typical size in the growth direction being much smaller than the typical size in the surface plane.

In the present work, we present the general analytical theory of the few-particle system confined in an asymmetric biconvex quantum lens and lowest optical resonances in this system, and also show the supposed realization of the generalized Kohn theorem. We present the results of direct measurements of optical absorption spectra in far-IR range in the MBE grown self-assembled Ge/Si quantum dot arrays with an increasing average number of holes in dots and discuss the applicability of the presented general theory to the Ge/Si quantum dots with a few holes inside.

## 2. Materials and Methods

The Ge/Si quantum dot structures were grown by MBE using the Sb-mediated technology described in detail in Refs [55,56]. We studied four structures with 10 layers of quantum dots separated with 15 nm Si barrier layers. Structures were grown by the same receipt but differ in the boron doping level in the δ-layer 5 nm below each QD layer. The nominal doping levels of the QDs in four structures were 6, 4, 2, and 0 holes per 1 QD (the last structure was undoped). The dot material is Ge_x_Si_1−x_ solid solution with Ge content about 60%. The effective mass of heavy holes in QDs was estimated as $\mu =xm_{Ge}+(1-x)m_{Si}$, where $m_{Ge}=0.33m_{0}$ and $m_{Si}=0.49m_{0}$ are the heavy hole masses for Ge and Si respectively [57]. The shape and average size of the QDs was determined with the XTEM and AFM pictures and can be estimated as lens-shaped islands with (2.75 ± 0.2) nm height and (14 ± 4) nm base.

The optical transmission spectra were measured with the Fourier-transform infrared spectrometer Bruker Vertex 80v (Bruker Optik GmbH, Ettlingen, Germany). Globar was used as a source of the broadband far-IR radiation. The liquid-helium-cooled Si bolometer was used as a detector of the radiation. The samples for optical measurements were prepared in multipass geometry [58] and mounted in liquid-nitrogen-cooled optical cryostat with polyethylene input and output windows. The overall accessible spectral range of the spectrometer with the Mylar beamsplitter was 100–500 cm^−1^ (approx. 10–60 meV). The spectral resolution was 16 cm^−1^.

In equilibrium transmission measurements of the doped samples the undoped sample was used as a reference. In addition, we also studied the far-IR photoresponse of undoped sample related to photoexcited holes under conditions of external interband optical excitation with CW YAG:Nd laser with frequency doubling, that was mechanically chopped at 90 Hz. In this experiment, the difference in far-IR optical transmission related to interband photoexcitation was measured at the chopping frequency with the SR830 Lock-In amplifier (Stanford Research Systems, Sunnyvale, CA, USA).

In order to check the correspondence of the nominal doping and the real average number of holes in the QDs, we have also measured the mid-infrared absorption spectra of all the structures in the spectral range corresponding to the valence band offset energy. We have observed the absorption peak close to 300 meV photon energy that corresponds to the optical transitions of holes from the QD ground state to the continuum above the barrier, that was also proved by the lateral mid-IR photoconductivity measurements [58]. The absorption coefficient related to these transitions is found to linearly increase with the increase of the nominal doping level, as was expected since in this spectral range the transition energy is much larger than the energy of the Coulomb attraction of the holes inside QD and the usual absorption cross-section can be introduced. Therefore, we have Ge/Si QD structures with a sequentially increasing number of holes in QD, i.e., there are charged quantum dots with the charge equal to several elementary charges *e*.

## 3. Theoretical Model for Asymmetric Biconvex Quantum Lens

To construct the theoretical model, we note those important provisions, which underlie the proposed theory and are implemented in the experiment:

(1) QD contains few-particle gas (particularly, gas of heavy holes);
(2) The effective mass of particles is scalar;
(3) The QD has specific geometry, which allows dividing the particle’s motion into “fast” and “slow”;
(4) The incident perturbation on the system is long-wavelength.

It is important to note that QDs in the experiment are MBE grown Ge dots in Si matrix with a deep potential well in the valence band for holes. Special attention should be paid to possible coupling of the heavy and light hole states in this case, as was shown in Ref. [59] for GaAs-based QDs. However, numerical estimations show that in case of Ge/Si QDs, the low-level QD states are formed mainly by the heavy hole states and the intermixing effects of the heavy and light hole states can be neglected due to both the small value of the light hole effective mass in Ge and the strong vertical confinement. As a result, one can consider the holes in QD as a heavy hole gas characterized by the scalar effective mass, non-interacting with light hole band, and thus one can write the multiparticle Hamiltonian with scalar effective mass μ.

**Figure 1:**
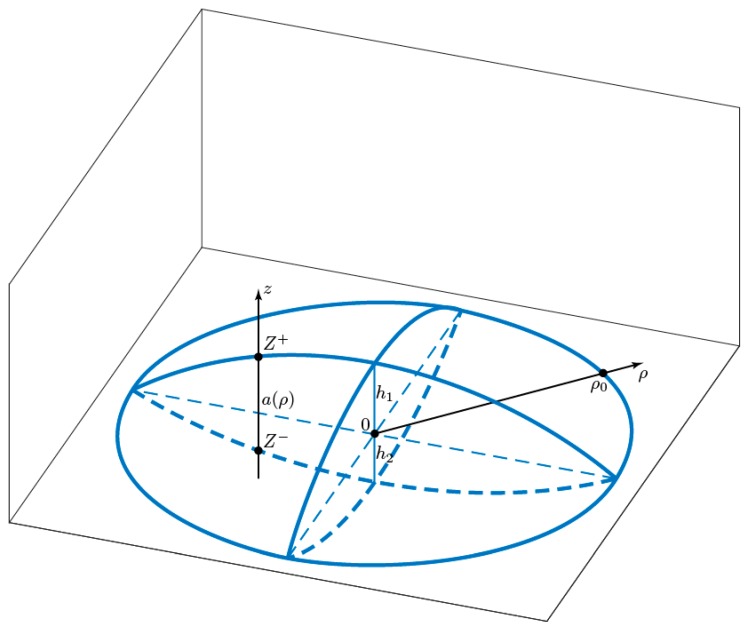
Schematics of the asymmetric biconvex quantum lens under consideration.

Let’s consider pair-interacting electronic gas, localized in asymmetric biconvex quantum lens (ABCQL), shown in Figure 1. The interparticle Coulomb interaction in *z* direction can be considered weak in comparison with the size quantization, taking into account the small thickness of the system in this direction, and therefore, we will consider the interaction operator between particles as two-dimensional:

(1) $${\hat{V}}_{\mathrm{int}}\left(1,2,\ldots ,N\right)=\sum_{i<j}\upsilon \left(\left|{\vec{\rho }}_{i}-{\vec{\rho }}_{j}\right|\right).$$

For the relatively low levels of charge carriers the confinement potential of QD can be approximated by a rectangular infinitely deep well:

(2) $$U_{conf}\left(\rho ,z\right)=\{\begin{array}{c} 0,M\in QD\\ \infty ,M\notin QD \end{array}.$$

Taking into account (1) and (2), the N-particle Hamiltonian of the system has the following form:

(3) $$\hat{H}\left(1,\ldots ,N\right)=\frac{1}{2\mu }\sum_{j=1}^{N}{\hat{P}}_{j}^{2}+\sum_{j=1}^{N}U_{conf}\left(j\right)+{\hat{V}}_{\mathrm{int}}\left(1,\ldots ,N\right).$$

We will not specify the type of interparticle interaction potential, and we only suppose the fact that it depends on the interparticle distance modulus only. The ABCQL’s finesse condition

(4) $$\left\{h_{1},h_{2}\right\}\ll \rho_{0}$$

makes it possible to use the adiabatic method for the description of the electronic or hole gas in such a system.

The description will be implemented in two stages:

- First we will consider single-particle states in ABCQL.
- On the basis of the single-particle model we will describe pair-interacting gas.

### 3.1. Single-Particle States in ABCQL

The Schrodinger equation for the particle, localized in ABCQL with confining potential (2), has the form:

(5) $$-\frac{\hbar^{2}}{2\mu }\left(\frac{1}{\rho }\frac{\partial }{\partial \rho }\left(\rho \frac{\partial }{\partial \rho }\right)+\frac{1}{\rho^{2}}\frac{\partial^{2}}{\partial \varphi^{2}}+\frac{\partial^{2}}{\partial z^{2}}\right)\psi \left(\rho ,\varphi ,z\right)+U_{conf}\left(\rho ,z\right)\psi \left(\rho ,\varphi ,z\right)=E\psi \left(\rho ,\varphi ,z\right).$$

The motion along the axis OZ occurs much faster than in the plane of the section XOY. According to the adiabatic approach, in this case the wave function of the system can be represented as a product

(6) $$\psi \left(\rho ,\varphi ,z\right)=f\left(\vec{\rho }\right)\chi \left(z;\rho \right),$$

where χ(z;ρ) describes one-dimensional motion along the axis OZ at a fixed value of the radial coordinate ρ, $f\left(\vec{\rho }\right)$ is the particle’s wave function in the plane of the section of ABCQL. By fixing the value ρ one can show that the particle carries out the motion along OZ, being in a rectangular infinitely deep quantum well with boundary points

(7) $$Z^{+}=\sqrt{R_{1}^{2}-\rho^{2}}+h_{1}-R_{1},$$

and

(8) $$Z^{-}=-\sqrt{R_{2}^{2}-\rho^{2}}-h_{2}+R_{2}.$$

Meanwhile, the thickness of the one-dimensional well will be equal to:

(9) $$a\left(\rho \right)=\sqrt{R_{1}^{2}-\rho^{2}}+\sqrt{R_{2}^{2}-\rho^{2}}+\left(h_{1}+h_{2}\right)-\left(R_{1}+R_{2}\right),$$

where $R_{1(2)}$ are radiuses of spheres, the intersection of which forms the surface of ABCQL.

The solution of the one-dimensional Schrödinger equation is the function

(10) $$\chi \left(z;\rho \right)=\sqrt{\frac{2}{a\left(\rho \right)}}\sin\frac{\pi n}{a\left(\rho \right)}\left(z+\sqrt{R_{2}^{2}-\rho^{2}}+h_{2}-R_{2}\right).$$

For the spectrum we will get

(11) $$E_{n}^{z}\left(\rho \right)=\frac{\pi^{2}\hbar^{2}n^{2}}{2\mu a^{2}\left(\rho \right)}\equiv U_{n}^{eff}\left(\rho \right),$$

where n is an axial quantum number.

According to the adiabatic approximation [46], the energy of the “fast” subsystem $E_{n}^{z}\left(\rho \right)$, which parametrically depends on the coordinate of the “slow” subsystem ρ, plays a role of the effective potential energy for the “slow” subsystem. Hence, for the wave function $f\left(\vec{\rho }\right)$ we will get the two-dimensional Schrodinger equation:

(12) $$-\frac{\hbar^{2}}{2\mu }\left(\frac{\partial^{2}}{\partial \rho^{2}}+\frac{1}{\rho }\frac{\partial }{\partial \rho }+\frac{1}{\rho^{2}}\frac{\partial^{2}}{\partial \varphi^{2}}\right)f\left(\vec{\rho }\right)+U_{n}^{eff}\left(\rho \right)f\left(\vec{\rho }\right)=E_{\rho }f\left(\vec{\rho }\right).$$

The Equation (12) with potential (11) doesn’t have an exact analytic solution, but if we assume that the particle is mainly localized around the geometric center of the QD, when $\rho \ll \rho_{0}$, then the potential function $U_{n}^{eff}\left(\rho \right)$ can be expanded in a Taylor series [46]:

(13) $$U_{n}^{eff}\left(\rho \right)\approx \frac{\pi^{2}\hbar^{2}n^{2}}{2\mu {\left(h_{1}+h_{2}\right)}^{2}}\left(1+\frac{R_{1}+R_{2}}{\left(h_{1}+h_{2}\right)\left(R_{1}R_{2}\right)}\rho^{2}\right)=\frac{\pi^{2}\hbar^{2}n^{2}}{2\mu {\left(h_{1}+h_{2}\right)}^{2}}+\frac{\mu \Omega_{n}^{2}\rho^{2}}{2},$$

where

(14) $$\Omega_{n}^{2}=\frac{R_{1}+R_{2}}{\mu^{2}{\left(h_{1}+h_{2}\right)}^{3}\left(R_{1}R_{2}\right)}\cdot \pi^{2}\hbar^{2}n^{2}.$$

In other words, the particle is in a two-dimensional parabolic well in the plane of the QD’s cross-section with a frequency $\Omega_{n}$. Finally, for the full energy of the system we will get:

(15) $$E_{n,n_{osc}}=\frac{\pi^{2}\hbar^{2}n^{2}}{2\mu {\left(h_{1}+h_{2}\right)}^{2}}+\hbar \Omega_{n}\left(n_{osc}+1\right),$$

where $n_{osc}$ is oscillator quantum number.

### 3.2. Multiparticle States in ABSQL

Let us turn to the many-particle problem. Taking into account strong quantization in the z direction, N-particle wave function can be presented in the form of the following product [44]:

(16) $$\Psi \left({\vec{r}}_{1},\ldots ,{\vec{r}}_{N}\right)=\chi_{n_{1},\ldots ,n_{N}}\left(z_{1}\left(\rho_{1}\right),\ldots ,z_{N}\left(\rho_{N}\right)\right)F\left({\vec{\rho }}_{1},\ldots ,{\vec{\rho }}_{N}\right),$$

where $F\left({\vec{\rho }}_{1},\ldots ,{\vec{\rho }}_{N}\right)$ is the wave function, characterizing the gas in the plane of the cross-section of ABCQL, and $\chi_{n_{1},\ldots ,n_{N}}\left(z_{1}\left(\rho_{1}\right),\ldots ,z_{N}\left(\rho_{N}\right)\right)$ is the wave function, characterizing the gas in z direction.

As the interparticle interaction in the z direction can be neglected, then the function $\chi_{n_{1},\ldots ,n_{N}}$ can be represented as a product of single-particle wave functions (10):

(17) $$\chi_{n_{1},\ldots ,n_{N}}\left(z_{1}\left(\rho_{1}\right),\ldots ,z_{N}\left(\rho_{N}\right)\right)=\prod_{j=1}^{N}\sqrt{\frac{2}{a\left(\rho_{j}\right)}}\sin\frac{\pi n_{j}}{a\left(\rho_{j}\right)}\left(z+\sqrt{R_{2}^{2}-\rho_{j}^{2}}+h_{2}-R_{2}\right).$$

The corresponding energy $E_{n_{1},\ldots ,n_{N}}^{z}\left(\rho_{1},\ldots ,\rho_{N}\right)$ has the form:

(18) $$E_{n_{1},\ldots ,n_{N}}^{z}\left(\rho_{1},\ldots ,\rho_{N}\right)=\sum_{j=1}^{N}\frac{\pi^{2}\hbar^{2}n_{j}^{2}}{2\mu a^{2}\left(\rho_{j}\right)}.$$

Based on (18), for the wave function $F\left({\vec{\rho }}_{1},\ldots ,{\vec{\rho }}_{N}\right)$ we will get the following equation:

(19) $$\left\{\sum_{j=1}^{N}\frac{1}{2\mu }\left({\hat{P}}_{xj}^{2}+{\hat{P}}_{yj}^{2}\right)+\sum_{j=1}^{N}\frac{\pi^{2}\hbar^{2}n^{2}}{2\mu a^{2}\left(\rho_{j}\right)}+\sum_{i<j}\upsilon \left(\left|{\vec{r}}_{i}-{\vec{r}}_{j}\right|\right)\right\}F\left({\vec{\rho }}_{1},\ldots ,{\vec{\rho }}_{N}\right)=EF\left({\vec{\rho }}_{1},\ldots ,{\vec{\rho }}_{N}\right).$$

If we consider few-particle gas, then this system will be localized around the geometric center of the QD due to the repulsion of the walls. Consequently, the effective potential energy (18) can be expanded in a Taylor series by analogy with single-particle case. As the size quantization along the *Z* axis is strong, then one can assume that all particles are in the ground states in this direction with $\Omega \equiv \Omega_{1}$, where

(20) $$\Omega \equiv \Omega_{1}=\frac{\pi \hbar }{\mu }{\left(\frac{R_{1}+R_{2}}{R_{1}R_{2}}\cdot \frac{1}{{\left(h_{1}+h_{2}\right)}^{3}}\right)}^{1/2}.$$

Thus, the hole gas in the plane of the section of ABCQL will be described by two-dimensional Hamiltonian

(21) $${\hat{H}}^{2D}=\frac{1}{2\mu }\sum_{j=1}^{N}\left({\hat{P}}_{xj}^{2}+{\hat{P}}_{yj}^{2}\right)+\frac{\mu \Omega^{2}}{2}\sum_{j=1}^{N}\left(x_{j}^{2}+y_{j}^{2}\right)+\sum_{i<j}\upsilon \left(\left|{\vec{r}}_{i}-{\vec{r}}_{j}\right|\right).$$

It is well known that the non-interacting part of Hamiltonian

(22) $${\hat{H}}_{0}^{2D}=\frac{1}{2\mu }\sum_{j=1}^{N}\left({\hat{P}}_{xj}^{2}+{\hat{P}}_{yj}^{2}\right)+\frac{\mu \Omega^{2}}{2}\sum_{j=1}^{N}\left(x_{j}^{2}+y_{j}^{2}\right),$$

can be exactly diagonalized and represented with the help of creation and annihilation operators:

(23) $${\hat{C}}_{x\left(y\right)}^{\pm }={\left(\frac{\mu \Omega }{2\hbar }\right)}^{1/2}\sum_{j=1}^{N}\left(x_{j}\left(y_{j}\right)\mp i\frac{{\hat{P}}_{x_{j}\left(y_{j}\right)}}{\mu \Omega }\right).$$

Hamiltonian in terms of creation and annihilation operators has the form:

(24) $${\hat{H}}_{0}^{2D}=\hbar \Omega \left({\hat{C}}_{x}^{+}{\hat{C}}_{x}^{-}+\frac{1}{2}\right)+\hbar \Omega \left({\hat{C}}_{y}^{+}{\hat{C}}_{y}^{-}+\frac{1}{2}\right).$$

A direct calculation shows that the following commutations take place:

(25) $$\left[{\hat{V}}_{\mathrm{int}},{\hat{C}}_{x}^{\pm }\right]=\left[{\hat{V}}_{\mathrm{int}},{\hat{C}}_{y}^{\pm }\right]=0.$$

(26) $$\left[{\hat{H}}_{0}^{2D},{\hat{C}}_{x}^{\pm }\right]=\pm \hbar \Omega {\hat{C}}_{x}^{\pm },\left[{\hat{H}}_{0}^{2D},{\hat{C}}_{y}^{\pm }\right]=\pm \hbar \Omega {\hat{C}}_{y}^{\pm }.$$

Based on (25) and (26) one can write the following relations:

(27) $$\left[{\hat{H}}^{2D},{\hat{C}}_{x}^{\pm }\right]=\left[\left({\hat{H}}_{0}^{2D}+{\hat{V}}_{\mathrm{int}}\right),{\hat{C}}_{x}^{\pm }\right]=\pm \hbar \Omega {\hat{C}}_{x}^{\pm },$$

and

(28) $$\left[{\hat{H}}^{2D},{\hat{C}}_{y}^{\pm }\right]=\pm \hbar \Omega {\hat{C}}_{y}^{\pm }.$$

Suppose $F_{n_{x},n_{y}}$ is an eigenfunction of the operator ${\hat{H}}^{2D}$. With the help of (25)–(28) one can show that ${\hat{C}}_{x}^{+}F_{n_{x},n_{y}}$ is also an eigenfunction of ${\hat{H}}^{2D}$. At that if $F_{n_{x},n_{y}}$ corresponds to the energy $E_{n_{x},n_{y}}$, then ${\hat{C}}_{x}^{+}F_{n_{x},n_{y}}$ will correspond to the energy $E_{n_{x},n_{y}}+\hbar \Omega$. The similar reasoning takes place for the operators ${\hat{C}}_{x}^{-},{\hat{C}}_{y}^{+},{\hat{C}}_{y}^{-}$. It is noteworthy that in the case of the Hamiltonian ${\hat{H}}_{0}^{2D}$ the following similar reasoning takes place too:

(29) $$\begin{array}{l} F_{n_{x},n_{y}}^{\left(0\right)}\to E_{n_{x},n_{y}}^{\left(0\right)}\\ {\hat{C}}^{+}F_{n_{x},n_{y}}^{\left(0\right)}\to E_{n_{x},n_{y}}^{\left(0\right)}+\hbar \Omega . \end{array}$$

Here $F_{n_{x},n_{y}}^{0}$ is the eigenfunction of the operator ${\hat{H}}_{0}^{2D}$.

In other words, the energy of the system under the influence of the operator ${\hat{C}}_{x}^{+}$ increases by ℏΩ both for pair-interacting and non-interacting gases.

Suppose now that the long-wave radiation is incident on the system perpendicular to the plane of the QD section:

(30) $${\hat{H}}_{1}=-e\left\{\sum_{j=1}^{N}{\vec{\rho }}_{j}\right\}\vec{E}\left(t\right),$$

where the electric field of a light wave with amplitude $\vec{E_{0}}$

(31) $$\vec{E}\left(t\right)=e^{-i\omega t}{\vec{E}}_{0}.$$

A direct calculation shows that

(32) $${\sum_{j=1}^{N}x_{j}\left(y_{j}\right)=\left(\frac{\hbar }{\mu \Omega }\right)}^{1/2}\left({\hat{C}}_{x(y)}^{+}+{\hat{C}}_{x(y)}^{-}\right).$$

Based on (30) and (32) it follows that if ${\hat{H}}_{1}$ acts on $F_{n_{x},n_{y}}^{\left(0\right)}$ from one side and on $F_{n_{x},n_{y}}$ from the other side, then in both cases, there will be resonant transitions with the same energy under the influence of long-wave radiation:

(33) ΔE=ℏΩ.

Consequently, the energy of the resonant transitions caused by the action of long-wave radiation does not depend on the interparticle interaction. Thus, the conditions for the realization of the generalized Kohn theorem in the system take place.

## 4. Experimental Results and Discussion

In order to observe the manifestation of the generalized Kohn theorem in real experiments, we selected the structure with Sb-mediated self-assembled Ge/Si quantum dots. This material system is uniquely suitable for such types of experiments in comparison with any quantum dot structures in an A^3^B^5^ material system.

First, the typical lattice vibration frequencies are in the same range as the frequencies of the confinement resonances Ω. The strong coupling of the charge carriers with the optical phonons in polar A^3^B^5^ materials makes the initial problem formulation radically different and leads to consideration of the polaron size quantization. However, in homeopolar Ge and Si materials the optical phonon coupling is negligible and one can expect the clear multi-particle effects in the quantization of the charge carriers.

Second, the large valence band offset in Ge/Si heterointerface leads to strong enough confinement of holes inside QDs. The typical lowest size resonance energies are about one order of magnitude smaller then the overall confinement energy. Together with the specific shape of these QDs with a large aspect ratio, it creates the conditions required for the adiabatic separation of the motion of holes in the directions of strong and weak confinement. It should be also noted, that the relatively large QDs density provided with the Sb-mediated MBE growth process simplifies the experimental measurements of the QDs optical response.

In our experiment, we directly studied the optical transmission spectra of the Ge/Si quantum dot structures with different doping level in the far-IR range corresponding to the expected resonant frequencies Ω.

**Figure 2:**
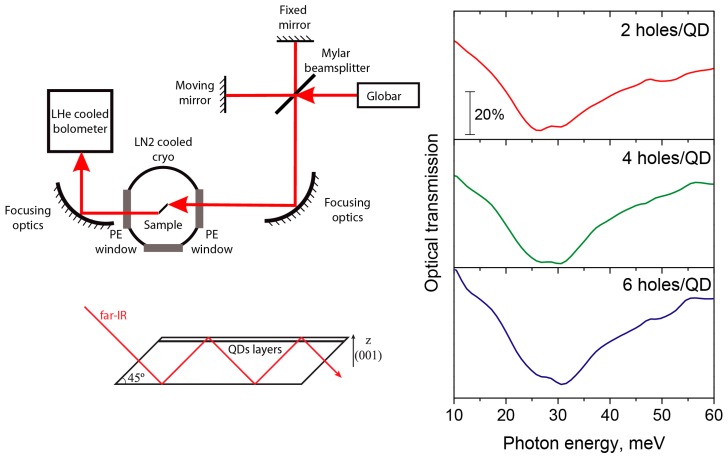
Experiment schematics and the measured far-IR transmission spectra of the Ge/Si quantum dot structures with different doping levels at 80 K. The labels at the curves indicate the nominal doping level.

Measured transmission spectra of doped QD samples are plotted in Figure 2 together with the experiment schematics. All doped samples demonstrate a broadened absorption peak with the center at about 30 meV. Moreover, the shape and position of the absorption peaks seems to be the same for the structures with nominal doping of 2, 4, and 6 holes per dot, or, in other words, independent of the number of holes in the QDs.

Let us discuss the application of the presented general theory to the Ge/Si QDs. First of all, the shape of the QDs can be described as a flat-convex-lens and the adiabatic separation (6) can be applied.

The far-IR transition energy is one order of magnitude smaller than the valence band offset that forms the real confinement potential, thus the approximation (2) can be used for confinement in the energy range of interest.

Also we should neglect the intermixing of the light and heavy hole states and consider only the heavy holes since they form the lowest states of size quantization. There are several works that show the coupling of the center-of-mass and relative motion due to the mixing of the basis states of the multiband Hamiltonian of the valence band (see, for example, ref. [60] and references therein). The most relevant work [59] describes the effect of the heavy and light hole coupling under the conditions of relatively weak vertical confinement and small enough splitting of the corresponding energy terms. However, in the case of Ge/Si QDs studied in the experiment, the simple estimations show more than 500 meV splitting of the heavy and light holes for largest dots in ensemble due to strong vertical confinement, which in turn leads to negligible contribution of the light hole states to the QD ground state. Taking into account the strain of the QD and surrounding matrix will result only in a change of the splitting of the heavy and light holes, since the shear components of the strain in similar QDs are found to be very small [61]. Thus, considering only a heavy-hole gas in QD is an assumption of the same order as an infinite-barrier approximation.

Next, in spite of the complicated distribution of the holes over the inhomogeneously broadened QD ensemble, at low temperatures the largest dots in the ensemble are expected to be occupied with holes, since both the Coulomb attraction and size quantization energy decrease with the increase in characteristic confinement length. The shape of the absorption band is determined by the number of dots in the ensemble with hole(s) inside having a certain size corresponding to the certain resonant frequency. It is a challenging task to calculate this number for the ensemble with size dispersion, because the QD ground state energy depends on both QD size and the number of holes inside. But, qualitatively, an increase in total number of holes in the system should lead to an increase in population of smaller dots with higher resonant energies. Thereby, we can attribute the weak broadening of the measured absorption band to the short-wavelength region with the increase of doping level to the increase in probability of population of smaller dots.

Therefore, we can now apply the presented general theory of long-wave absorption by a hole gas, localized in ABCQL, to the plane-convex quantum lens, that represents the shape of the Ge/Si quantum dots studied in experiment, with the following parameters: *h*_1_ = 2.95 nm, *h*_2_ = 0, $\rho_{0}$ = 9 nm, R = (*h*_1_^2^ + $\rho_{0}$^2^)/2*h*_1_ = 15.2 nm, and μ = 0.39*m*_0_, corresponding to the real size of the largest dots in the ensemble.

From the general formula (20) for resonant frequencies Ω in the case of a plane-convex lens, we can immediately write:

(34) $$\Omega =\frac{\pi \hbar }{\mu }{\left(\frac{1}{R_{1}h_{1}^{3}}\right)}^{1/2}.$$

For the parameters given above, the theoretically calculated energy (based on (34)) gives the value:

(35) $$\hbar \Omega_{\mathrm{theor}}≃31\mathrm{meV}.$$

In turn, the experimentally measured value of the energy of the resonant transitions in the center of the broadened absorption band that, again, corresponds to large dots, is

(36) $$\hbar \Omega_{\exp}≃30\mathrm{meV}.$$

Thus, there is very good consistency between the experimentally found and theoretically calculated values of the resonant transition energies.

Therefore, we observe the single-particle-like optical transitions in the multi-particle system with the pair Coulomb interaction.

**Figure 3:**
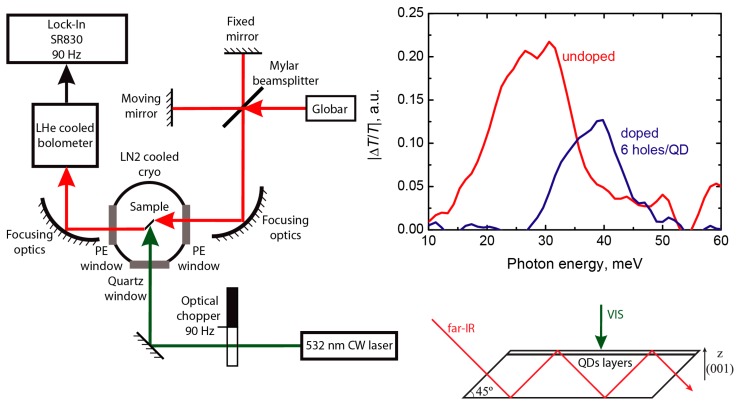
Experiment schematics and the measured spectra of the change of far-IR transmission due to external interband photoexcitation at 80 K. Data are plotted for undoped Ge/Si quantum dot structure (red curve) and doped structure (blue curve) with nominal doping of 6 holes/dot.

Let us now consider the far-IR photoresponse of the undoped QDs related to the photoexcited holes created by the external interband excitation with a 532 nm laser radiation. In this case, the interband excitation creates electron-hole pair in the silicon. The holes can be captured to the quantum dots, but electrons can only be localized at the weak potential wells at the Si side of the QD heterointerface induced by the built-in strain (see, for example reference [62] and references therein). Figure 3 shows the experiment schematics and the spectral dependence of the relative change ΔT/T of sample transmission induced by the interband photoexcitation both for undoped and doped structures, which actually gives the photoinduced optical absorption curve. The photoresponse of the undoped sample caused by the occupation of the QDs with nonequilibrium holes is very similar to the equilibrium optical transmission spectra and demonstrates the same broadened resonance due to optical far-IR transition to the lowest excited state of the multi-particle system.

The photoresponse of the doped sample demonstrates only the contribution of the quantum dots that are not occupied with resident carriers, since the addition of the non-equilibrium photoexcited hole to the dot that already have a hole inside doesn’t provide any change in the far-IR photoresponse due to the generalized Kohn theorem. As we have mentioned above, in doped samples the largest dots with lower resonance energies Ω are occupied with resident holes. As a result, only the response of small dots with higher resonance energies Ω contributes to the measured spectra. Consequently, there is a noticeable “blue shift” of the photoiduced far-IR absorption spectra of doped sample with respect to undoped one. However, the overall spectral shape of the photoiduced absorption peak of doped quantum dots can also be influenced by the difference in the capture rate of holes to the charged and empty QDs.

## 5. Conclusions

Experimental results suggesting feasibility of the implementation of the generalized Kohn theorem for a gas of heavy holes in the lens-shaped Ge/Si QD are presented. In the framework of the adiabatic method, it was theoretically shown that the specific geometry of a QD leads to the formation of a two-dimensional parabolic confining potential in the sectional plane of the ABCQL. Considering the interaction between heavy holes as paired and depending only on the modulus of distance between particles, the single-particle nature of the far-IR absorption is shown. For this QD model, the analytical expression for the resonant absorption frequencies gives a good quantitative agreement with the results of the far-IR spectroscopy.
